# Impact of sleep and psychological flexibility on young adults’ physical and mental well-being

**DOI:** 10.1038/s41598-026-53737-4

**Published:** 2026-05-20

**Authors:** Miho Ishii, Miyu Hakoda, Isa Okajima

**Affiliations:** 1 Senzoku Stress Coping Support Office, 2-29-4, Kitasenzoku, Ota, Tokyo, Japan; 2 Department of Neuropsychiatry, Kyorin University School of Medicine, 6- 20-2, Shinkawa, Mitaka, Tokyo Japan; 3 Noda Campus, Azusa Daiichi High School, 405-1, Noda, Noda, Chiba Japan; 4 Behavioral Sleep Medicine and Sciences Laboratory, Department of Psychological Counseling, Faculty of Humanities, Tokyo Kasei University, 1-18-1, Kaga, Itabashi, Tokyo Japan

**Keywords:** Insomnia, Circadian rhythm, Sleep debt, Psychological flexibility, Mental well-being, Physical well-being, Health care, Neuroscience, Psychology, Psychology

## Abstract

Young adults frequently report sleep issues, especially insomnia, delayed sleep phase, and sleep debt (Trisleep), with each of these contributing to distinct physical and mental well-being issues. Psychological flexibility (Hexaflex) has likewise been shown to relate to both physical and mental well-being. This study explored the Trisleep and Hexaflex among young adults, analyzing their association with physical and mental well-being. All questionnaire data were collected online from 525 university students in Japan (*M*_*age*_ = 19.96 ± 1.42 years). In a cluster analysis, four Trisleep types (good sleep, sleep–wake rhythm problems, sleep debt, and compound problems) and four Hexaflex types (flexibility, medium-flexibility, mild-flexibility, and inflexibility), differentiated by overall levels rather than distinct features were identified. Trisleep types (Deviance = 75.26, *p* <  0.0001), especially compound sleep problems, strongly affected physical well-being (*t* = − 6.97, *p* < 0.0001). The interaction between Trisleep and Hexaflex types significantly predicted mental well-being (Deviance = 20.81, *p* = 0.033). Across all sleep types, the Inflexibility type consistently had a stronger negative effect on mental well-being than the Flexibility type (*p*s < 0.05). The findings underscore the importance of addressing both biological and psychological factors to enhance physical and mental well-being, with targeted interventions for sleep and psychological flexibility, potentially yielding effective outcomes for young adults.

**Supplementary Information:**

The online version contains supplementary material available at 10.1038/s41598-026-53737-4.

## Introduction

 Sleep problems are common during adolescence and up to young adulthood, with three primary issues frequently identified: sleep quality, sleep rhythm, and sleep quantity^1^. Sleep quantity refers to the total amount of sleep an individual requires and can be expressed in terms of sleep debt. Sleep-wake rhythm describes the chronotype such as morningness and eveningness, and the pattern of sleep–wake timing, with regularity and alignment with social rhythms considered optimal. Finally, sleep quality refers to a broad construct encompassing nocturnal symptoms such as difficulty in initiating and maintaining sleep, and daytime symptoms such as daytime sleepiness. Each of these factors contributes to distinctive physical and mental well-being issues. Sleep debt has been associated with increased physical pain, decreased academic performance, and a higher likelihood of depressive mood^2–5^. Delayed sleep-wake phase is often linked to depressive symptoms^6^, while insomnia tends to manifest as headaches, abdominal pain, or depressive mood^7–9^. The association between insomnia, pain, and depression is suggested to share a common neurophysiological basis, which is referred to as the pain–insomnia–depression syndrome (PIDS)^10^. The PIDS includes common factors such as hippocampal atrophy, central nervous system sensitization, and the orexin system^10^.

Prior research has consistently shown that sleep disorders significantly affect both physical and mental well-being; however, there are no comprehensive studies examining these triangular sleep issues (we named them “Trisleep” for convenience), simultaneously in young adults.

An evidence-based approach to addressing physical symptoms and mental health concerns is acceptance and commitment therapy (ACT), which is based on the assumption that psychological vulnerability, referred to as psychological inflexibility, underlies human psychopathology^11^. Psychological flexibility is defined as the tendency to notice internal and external experiences, consider the situational context, and engage in behaviors that promote acceptance, persistence, adaptation, and movement toward valued goals^12^. Six core processes, collectively known as the “Hexaflex,” are used to assess the degree of psychological flexibility: acceptance (openly allowing internal experiences), cognitive defusion (gaining distance from thoughts rather than being dominated by them), self-as-context (a stable sense of self as an observer of experiences), values (clarification of personally meaningful life directions), committed action (taking sustained, effective action guided by values), and mindfulness (present-moment awareness with nonjudgment). Some studies have examined how each of these six core processes is related to different symptoms. Regarding physical symptoms, an association between each component of psychological inflexibility and chronic pain has been previously reported. For example, mindfulness has been shown to alleviate pain^13^ and one proposed mechanism is that it reduces pain catastrophizing, thereby decreasing the perception of pain^14^. In addition, studies examining the Hexaflex in relation to pain simultaneously have suggested that experiential avoidance (i.e., non-acceptance) shows a weak association with pain^15^. Moreover, experiential avoidance and cognitive fusion (i.e., cognitively non-defusion) have been thought to contribute to emotional distress and lower quality of life, highlighting the importance of addressing them to reduce depression and anxiety^16–18^. Research has shown that psychological flexibility is associated with well-being in patients with obesity^19^. These findings suggest that each component of psychological flexibility influences physical and psychological well-being.

Although correlations between multidimensional psychological flexibility and chronic pain have been demonstrated^15^, no studies have examined the relationship between the overall Hexaflex and physical and mental well-being. While both psychological flexibility and sleep problems have been individually implicated in mental and physical well-being, prior research has primarily examined these constructs in isolation. Recent studies have suggested an association between psychological flexibility and sleep problems^20,21^. Thus, it remains unclear how sleep problems and psychological flexibility may interact to influence well-being^22^ or how their combined examination may reveal patterns not detectable when each is assessed separately. Considering that each construct independently contributes to well-being, investigating their combined and distinct effects is warranted. Apropos this, the present study aimed to (1) examine the characteristics of Trisleep and Hexaflex using each variable to understand sleep problems and psychological inflexibility in young adults, and (2) explore how these comprehensive categories associate with young adults’ physical and mental well-being. Based on prior evidence, we hypothesized that both sleep problems and psychological flexibility would affect physical and mental well-being; in particular, individuals with both issues may exhibit the poorest well-being.

## Methods

### Participants

This study was conducted in August 2023 in Japan. The participants were university students randomly recruited via the internet survey company, Cross Marketing, Inc. Each respondent was assigned one ID by the company. Responses were collected anonymously. They received points (the point value is confidential) as a reward from the company. Participants’ ages ranged from 18 to 22 years. The study included only university students; students with other educational levels (e.g., high school and vocational school) were excluded. Overall, 600 completed responses were obtained. Among the respondents, 525 university students (men = 101, women = 414, prefer not to say = 10; mean age: 19.96 ± 1.42 years), who had completed the questionnaire and had a sleep debt index (SDI) score of zero or more, were selected and analyzed, based on previous research^23^. Informed consent in writing was acquired from each participant. The study was conducted in accordance with the principles of the Declaration of Helsinki and approved by the Ethics Committee of Tokyo Kasei University (Approval No. R5-14).

### Measurements

#### Demographic information

Participants were asked about their age, sex, and educational level to identify university students most suited for the study.

#### Somatic symptom scale-8 (SSS-8)

The study used the SSS-8 to assess participants’ physical well-being^24,25^. This self-administered scale included eight items addressing physical symptoms such as stomatic or bowel problems and back pain. Each item was scored on a 5-point Likert scale (0–4), with total scores ranging from 0 to 32; higher total scores indicated worse somatic symptoms. Internal consistency of the scale was reported as acceptable, with a Cronbach’s alpha of 0.86^24^.

#### Subjective well-being scale (SWBS)

This study used the SWBS to assess mental well-being^26^. This self-administered scale consists of 15 items, each scored on a 4-point Likert scale (1–4), with total scores ranging from 15 to 60. The higher the overall score, the greater the level of well-being. Internal consistency of the scale was reported as acceptable, with a Cronbach’s alpha of 0.84^26^.

#### Sleep problems

##### Athens insomnia scale (AIS)

This study employed AIS to assess insomnia^27^. The self-administered scale comprises 8 items evaluating sleep quality over the past month, with each item scored on a 4-point Likert scale (0–3). The higher the total score (range: 0–24), the more severe the insomnia, and a score of 6 or higher was considered indicative of suspected insomnia. Internal consistency of the scale was reported as acceptable, with a Cronbach’s alpha of 0.88^27^.

##### Biological rhythms interview of assessment in neuropsychiatry (BRIAN)

This study used the BRIAN to assess delayed sleep-wake phase symptoms^28^. It is an interviewer-administered instrument that examines five domains—sleep, sociability, activities, eating, and biological rhythms—and comprises 21 questions related to delayed sleep–wake phase disorders^28^. The study used a self-administered questionnaire in which each item is scored on a 4-point Likert scale (1–4); total scores range from 21 to 84 (cut-off point: 40), with higher scores indicating greater disruption of circadian rhythms. A total score of 40 or higher was considered indicative of suspected delayed sleep phase syndrome. The higher the score, the greater disturbance of the biological rhythm. Internal consistency of the scale was reported as acceptable, with a Cronbach’s alpha of 0.82^28^.

##### Sleep debt index (SDI)

This study used the SDI to assess sleep debt^23^. This self-administered scale consists of three questions: Please tell us about your sleep patterns over the past month: (1) How long do you get nocturnal sleep on a weekday? (2) How long do you sleep on weekends? (3) Considering your own “feeling best of performance” rhythms, for how long would you sleep if you were free for an entire day? The SDI score is calculated as the discrepancy between self-reported ideal and real sleep time. Previous research has shown that when actual sleep time falls more than two hours below one’s ideal sleep duration, it negatively affects daytime sleepiness, productivity, and negative emotions^23^.

#### Psychological flexibility

##### Cognitive fusion questionnaire (CFQ)

The CFQ was used to evaluate cognitive fusion, the process by which thoughts and reality are confused^29^. The self-administered scale includes 13 items that investigate two domains: “cognitive fusion” and “cognitive defusion.” This study used only the “cognitive defusion” factor (4 items). Each item is scored on a 7-point Likert scale (1–7), with total scores ranging from 13 to 91 and scores for “cognitive defusion” ranging from 4 to 28. The higher the score, the greater the cognitive defusion. Internal consistency of the scale was reported as acceptable, with a Cronbach’s alpha of 0.68^29^.

##### Values of younger ages scale (VOYAGE)

This study utilized the Voyage to assess valued living^30^. This self-administered scale comprises 15 items across two domains: Clarification of Value and Commitment (10 items) and Continuation of Avoidance (5 items). The study used only the “Clarification of Value and Commitment” domain to assess clarity or lack of it in values and commitment of psychological flexibility. Each item is scored on a 4-point Likert scale (0–3), with total scores ranging from 0 to 30; the higher the score, the greater the clarity of values and commitment. Internal consistency of the scale was reported as acceptable, with a McDonald’s ω of 0.89 in university students^30^.

##### Three senses of the selves questionnaire (TSSQ)

The TSSQ was used to assess the three senses of the selves^31^. This self-administered scale comprises 33 items across four domains: “acting actively and flexibly in the world,” “conceptualized self,” “distancing from private events,” and “feeling the present moment.” In this study, “distancing from private events (3 items)” and “feeling the present moment (4 items)” domains were used to assess self-as-context and contact with the present moment (i.e., mindfulness) aspects of psychological flexibility. Each item is scored on a 7-point Likert scale (1–7), with scores ranging from 3–21 for “distancing from private events,” and 4–28 for “feeling the present moment”; the higher the score, the greater the self-as-context and contact with the present moment. Internal consistency of ‘distancing from private events’ and ‘feeling the present moment’ was reported as acceptable (Cronbach’s α = 0.78 and 0.72, respectively)^31^.

##### Acceptance process questionnaire (APQ)

This study used the APQ^32^ to calculate acceptance, which is the willingness to be open, receptively flexible, and non-judgmental toward ever-changing experiences. This self-administered scale comprises 13 items, examining four domains; the middle- or long-term factors are “expanding behavioral repertoire” and “being receptive to the real world,” while behavioral content factors are “making a choice not to avoid private events,” and “stopping reactions.” Each item is scored on a 7-point Likert scale (0–6), with total scores ranging from 0 to 78; higher scores indicate greater acceptance. Internal consistency of the scale was reported as acceptable, with a Cronbach’s alpha of 0.86^32^. The reliability and validity of each scale indicate in Supplementary 1.

### Statistical analysis

A hierarchical cluster analysis was conducted using Ward’s method to classify the Trisleep and Hexaflex types. For the Trisleep type, the AIS, SDI, and BRIAN scales were used to access the three dimensions of sleep quality, quantity, and biological rhythm, respectively. For the Hexaflex type, Voyage, TSSQ, CFQ, and APQ, six dimensions were value and commitment, self-as-context and mindfulness, cognitive defusion, and acceptance, respectively. However, because the Voyage scale has a one-factor structure, values and commitment were treated as a single construct. Subsequently, to visually clarify the characteristics of each cluster of Trisleep and Hexaflex types, the z-score for each scale were converted to deviation values and a radar chart was created.

Furthermore, generalized linear models (GLMs) were used to compare the impacts of Trisleep and Hexaflex types on participants’ physical and mental well-being. A GLM of gamma distribution with a log-link function was used to account for bias in the distributions of physical (SSS-8) and mental (SWBS) well-being. Given that the SSS-8 is a 5-point scale that includes 0 and is not suitable for gamma distribution, it was converted into a 5-point scale of 1–5 only during the analysis of GLM. The fixed effects were Trisleep clusters, Hexaflex clusters, and their interactions, and the dependent variables were SSS-8 and SWBS. A one-factor analysis of variance was conducted using a χ2 distribution to determine the difference in deviance between the models, including the factor to be tested (full model) and the model excluding the factor. Multiple comparisons were then conducted using a cluster of healthy participants as the criterion in the model, in which the difference in the level of deviance was significant. After examining the correlation coefficients for each of the explanatory variables (sleep and psychological flexibility) and the objective variables (physical and psychological well-being) from previous studies, the correlation coefficient for the combination with the lowest (sleep debt and well-being) was used to calculate the effect size (f2). Thereafter, with each of the two predictors, a significance level of 0.05, a power of 0.80, and an expected effect size of f² = 0.02^33^, the required sample size was estimated to be ≥ 484. Accounting for exclusions from the analysis, the required sample size was set at 600.

All statistical analyses were performed using the R Studio version 2023.12.1 Build 402. The R packages stats^34^, psych^35^, multcomp^36^, emmeans^37^, pwr^38^ were also used. The analysis of sex differences in the scales showed no significant differences.

## Results

### Classification of Trisleep and Hexaflex clusters

Table 1 presents the demographic data, means, and standard deviations (SDs) for each scale.

**Fig. 1:**
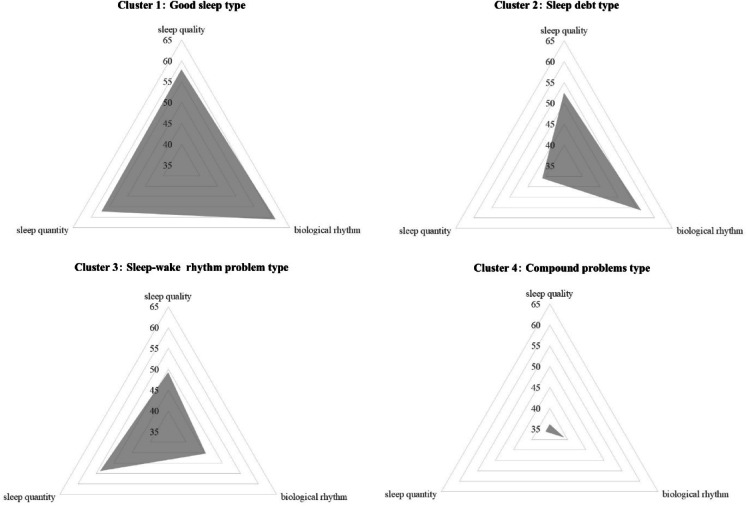
The result of cluster analysis of sleep problems. The variables of sleep quality were Athens Insomnia Scale (AIS), biological rhythm was Biological Rhythms Interview of Assessment in Neuropsychiatry (BRIAN), and sleep quantity was Sleep Debt Index (SDI).

For Trisleep, four clusters were identified: Cluster 1 was named “good sleep type,” characterized by low values of all the scales (*n* = 125); Cluster 2 was named “sleep debt type,” characterized by high values of SDI (*n* = 86); Cluster 3 was named “sleep-wake rhythm problem type,” characterized by high values of BRIAN (*n* = 239); and Cluster 4 was named “compound problems type,” characterized by high values of all the scales (*n* = 75) (Fig. 1). The results of the hierarchical cluster analysis based on Trisleep variables are presented in Supplementary 2.

**Table 1:** Demographic data and descriptive statistics of scales.

Valuables	Mean (SD)	*N* (%)
Age	19.96 (1.42)	
Sex		Men = 101 (19.24)
		Women = 414 (78.86)
		PNS = 10 (1.90)
AIS	5.26 (4.49)	
< 6 points		312 (59.43)
≥ 6 points		213 (40.57)
BRIAN	40.95 (12.61)	
< 40 points		249 (47.43)
≥ 40 points		276 (52.57)
SDI	1.19 (1.15)	
< 1 h		247 (47.05)
1–2 h		165 (31.43)
≥ 2 h		113 (21.52)
CFQ-cd	13.25 (4.84)	
VOYAGE-cvc	13.95 (7.20)	
TSSQ-dp	10.74 (3.82)	
TSSQ-fp	14.02 (4.48)	
APQ	32.97 (13.60)	
SSS-8	14.81 (6.78)	
SWBS	38.60 (8.03)	

AIS; Athens Insomnia Scale, APQ; Acceptance Process Questionnaire, BRIAN; Biological Rhythms Interview of Assessment in Neuropsychiatry, CFQ-cd; Cognitive Fusion Questionnaire-cognitive defusion subscale, PNS; prefer not to say, SD; Standardized deviation. SDI; Sleep debt index. SSS-8; Somatic Symptom Scale-8, SWBS; Subjective Well-Being Scale, TSSQ-dp; Three Senses of the Selves Questionnaire-distancing from private events subscale, TSSQ-fp; TSSQ-feeling the present moment subscale, VOYAGE-cvc; Values of Younger Ages Scale-clarification of value and commitment subscale.

**Fig. 2:**
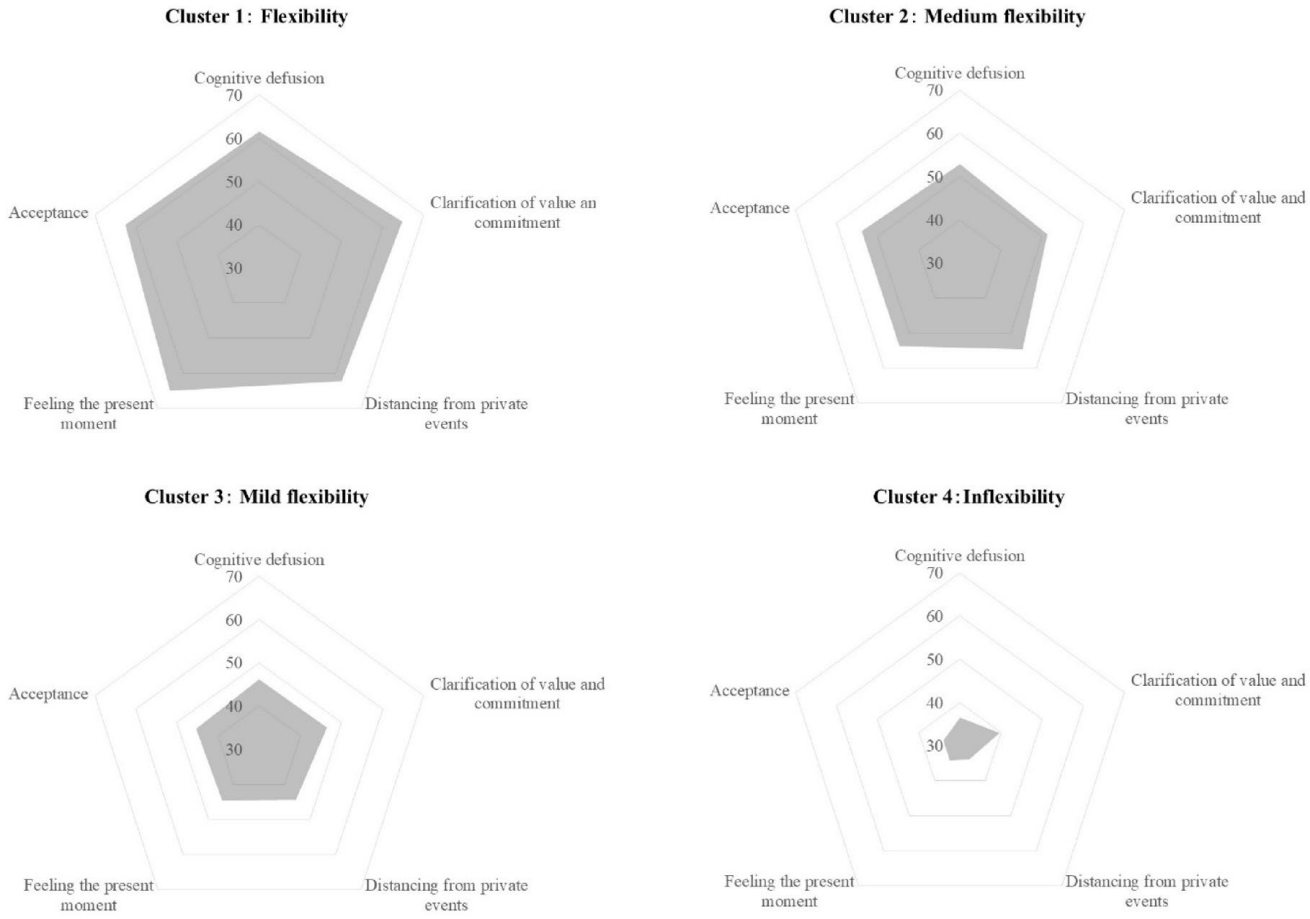
The result of cluster analysis of psychological flexibility. The variables of cognitive defusion were a domain of Cognitive Fusion Questionnaire (CFQ), Clarification of value and commitment was a domain of Values of Younger Ages scale (Voyage), distancing from private events and feeling the present moment were domains of Three Senses of the Selves Questionnaire (TSSQ), and acceptance was total score of Acceptance Process Questionnaire (APQ).

For Hexaflex, four clusters were identified and classified based on the total scores on each scale. Cluster 1 was named “flexibility” (*n* = 56), Cluster 2 “medium flexibility” (*n* = 236), Cluster 3 “mild flexibility” (*n* = 185), and Cluster 4 “inflexibility” (*n* = 48) (Fig. 2). The results of the hierarchical cluster analysis based on Hexaflex variables are presented in Supplementary 3. The means and SDs of each variable for each cluster are presented in Table 2.

**Table 2:** Means and standard deviations for each variable by cluster for Trisleep and Hexaflex.

	Trisleep				Hexaflex				
	Cluster 1: Good sleep type	Cluster 2: Sleep debt type	Cluster 3: Sleep-wake rhythm problem type	Cluster 4: Compound problems type	Cluster 1: Flexibility		Cluster 2: Medium flexibility	Cluster 3: Mild flexibility	Cluster 4: Inflexibility
N	125	86	239	75	56		236	185	48
AIS	1.69 (1.89)	4.13 (2.86)	5.59 (3.59)	11.45 (4.83)	4.48 (4.33)		5.45 (4.87)	5.42 (3.98)	4.63 (4.49)
BRIAN	27.06 (4.72)	32.99 (5.73)	46.74 (9.03)	54.76 (9.35)	37.89 (13.93)		41.14 (12.54)	42.83 (12.08)	36.35 (11.86)
SDI	0.36 (0.37)	2.22 (0.84)	0.75 (0.60)	2.78 (1.27)	1.17 (1.11)		1.11 (1.03)	1.24 (1.18)	1.40 (1.57)
CFQ-cd	13.37 (5.31)	13.14 (4.97)	13.39 (4.52)	12.72 (4.94)	18.86 (4.62)		14.66 (3.69)	11.44 (3.84)	6.75 (2.72)
VOYAGE- cvc	14.58 (8.75)	14.31 (7.33)	18.83 (6.46)	12.88 (6.36)	24.55 (4.00)		14.92 (5.67)	11.43 (6.26)	6.52 (4.96)
TSSQ-dp	10.98 (4.01)	10.83 (4.16)	10.58 (3.51)	10.73 (4.08)	15.45 (3.13)		12.52 (2.55)	8.65 (2.09)	4.52 (1.68)
TSSQ-fp	14.74 (4.95)	14.03 (4.62)	13.92 (4.22)	13.15 (4.20)	20.77 (2.97)		15.72 (2.80)	11.66 (2.56)	6.90 (2.51)
APQ	33.72 (14.33)	34.70 (13.73)	32.28 (12.97)	31.89 (14.20)	50.09 (11.77)		38.28 (8.37)	26.64 (9.19)	11.25 (9.50)
SSS-8	2.85 (4.57)	4.74 (5.97)	7.77 (6.34)	12.72 (7.13)	7.34 (8.38)		6.59 (6.60)	7.16 (6.42)	5.92 (7.05)
SWBS	39.99 (9.22)	41.40 (6.43)	38.72 (7.09)	36.08 (8.37)	46.13 (6.29)		39.27 (7.05)	36.03 (7.75)	34.76 (8.39)

AIS; Athens Insomnia Scale, APQ; Acceptance Process Questionnaire, BRIAN; Biological Rhythms Interview of Assessment in Neuropsychiatry, CFQ-cd; Cognitive Fusion Questionnaire-cognitive defusion subscale, SD; Standardized deviation. SDI; Sleep debt index. SSS-8; Somatic Symptom Scale-8, SWBS; Subjective Well-Being Scale. TSSQ-dp; Three Senses of the Selves Questionnaire-distancing from private events subscale, TSSQ-fp; TSSQ-feeling the present moment subscale, VOYAGE-cvc; Values of Younger Ages Scale-clarification of value and commitment subscale.

### Impacts of both Trisleep and Hexaflex on physical well-being

A GLM of gamma distribution with a log-link function was used to explore the differences in physical well-being between the Trisleep and Hexaflex clusters (Table 3). Deviance analysis was performed to verify the difference in deviance between the full model and the model excluding each factor. Significant differences were found in the deviance analysis, excluding the Trisleep cluster (Deviance = 75.262, *p* < .0001). The results of multiple comparisons using the good sleep cluster as the criterion in the interaction model showed that the types of sleep debt (t =-8.498, *p* = .048), sleep–wake rhythm (t =-5.677, *p* < .0001), and compound problem (t =-6.971, *p* < .0001) significantly negatively affected physical well-being. This result showed that participants in these sleep problem clusters had lower physical well-being compared to those in the good sleep cluster.

**Table 3:** Effects of characteristics of Trisleep and Hexaflex on physical well-being.

Physical well-being	Deviance	df	Emmean (95%CI)	SE	*t*	*p*	Contrasts
Null	99.249					-	
Hexaflex	98.844	3				0.484	
Trisleep	75.262	3				< 0.0001	
Hexaflex*Trisleep	74.230	9				0.714	
Trisleep characteristics							
1: Good sleep		509	2.39 (2.30–2.47)	0.042	-2.591	-	-
2: Sleep debt		509	2.56 (2.46–2.66)	0.052	-8.498	0.048	1 < 2
3: Sleep-wake rhythm		509	2.77 (2.70–2.84)	0.036	-5.677	< 0.0001	1 < 3
4: Compound problems		509	3.01 (2.89–3.13)	0.061	-6.971	< 0.0001	1 < 4

df; Degrees of Freedom, Emmean; Estimated Marginal Mean, p; p-value, SE; Standard Error, t; t-value, 95%CI; 95% Confidence Interval.

### Impacts of both Trisleep and Hexaflex on mental well-being

A GLM of gamma distribution with a log-link function was used to examine the differences in mental well-being between Trisleep and Hexaflex clusters (Table 4). Deviance analysis was performed to verify the difference in deviance between the full model and the model excluding each factor. Significant differences were found in the deviance analysis, excluding the Hexaflex cluster (deviance = 22.831, *p* < .0001), Trisleep cluster (deviance = 21.458, *p* < .0001), and their interactions (deviance = 20.809, *p* = .0326).

Multiple comparisons were subsequently conducted using a cluster of healthy participants (i.e., good sleep type or flexibility type) as criteria in the interaction model. In the good sleep type, the estimated marginal means (EMMs) for the mild flexibility (t = 3.951, *p* = .001) and inflexibility clusters (t = 6.530, *p* < .0001) were significantly lower affected to mental well-being, compared with the flexibility type. Comparing the EMMs for each psychological flexibility type, the medium flexibility (t = 2.588, *p* = .049) and mild flexibility (t = 3.905, *p* = .001) were shown to negatively affect mental well-being in sleep debt type; medium flexibility (t = 3.227, *p* = .007), mild flexibility (t = 4.766, *p* < .0001), and inflexibility (t = 4.158, *p* = .0002) negatively affected mental well-being in sleep–wake rhythm problem type; and mild flexibility (t = 3.513, *p* = .003) and inflexibility type (t = 2.795, *p* = .028) negatively affected mental well-being in compound problem type.

Similarly, comparing the EMMs for each sleep type, good sleep type, sleep–wake rhythm problem (t = 3.287, *p* = .006) and compound problem (t = 5.364, *p* < .0001) negatively affected mental well-being in medium flexibility; compound problem (t = 3.765, *p* = .001) negatively affected mental well-being mild flexibility; and sleep debt problem (t =-3.033, *p* < .014) negatively affected mental well-being in the inflexibility type.

**Table 4:** Characteristics of Trisleep and Hexaflex on mental well-being.

Mental well-being	Deviance	df	Emmean (95%CI)	SE	*t*	*p*	Contrasts
Null	26.396					-	
Hexaflex	22.831	3				< 0.0001	
Trisleep	21.458	3				< 0.0001	
Hexaflex*Trisleep	20.809	9				0.0326	
Good sleep							
1: Flexibility		509	3.86 (3.78–3.95)	0.042	-	-	-
2: Medium		509	3.76 (3.71–3.81)	0.025	2.077	0.162	-
3: Mild		509	3.65 (3.59–3.72)	0.031	3.951	0.001	1 > 3
4: Inflexibility		509	3.42 (3.32–3.53)	0.052	6.530	< 0.0001	1 > 4
Sleep debt							
1: Flexibility		509	3.88 (3.77–3.99)	0.057	-	-	-
2: Medium		509	3.71 (3.65–3.77)	0.031	2.588	0.049	1 > 2
3: Mild		509	3.61 (3.54–3.68)	0.036	3.905	0.001	1 > 3
4: Inflexibility		509	3.67 (3.55–3.80)	0.063	2.416	0.075	-
Sleep-wake rhythm problem							
1: Flexibility		509	3.81 (3.73–3.90)	0.043	-	-	-
2: Medium		509	3.66 (3.62–3.69)	0.018	3.227	0.007	1 > 2
3: Mild		509	3.58 (3.55–3.62)	0.020	4.766	< 0.0001	1 > 3
4: Inflexibility		509	3.55 (3.46–3.64)	0.045	4.158	0.0002	1 > 4
Compound problems							
1: Flexibility		509	3.77 (3.62–3.92)	0.077	-	-	-
2: Medium		509	3.54 (3.47–3.60)	0.033	2.795	0.028	1 > 2
3: Mild		509	3.47 (3.40–3.54)	0.036	3.513	0.003	1 > 3
4: Inflexibility		509	3.53 (3.40–3.66)	0.067	2.383	0.082	-
Flexibility							
1: Good sleep		509	3.86 (3.78–3.95)	0.042	-0.198	0.997	-
2: Sleep debt		509	3.88 (3.77–3.99)	0.057	1.026	0.734	-
3: Sleep-wake rhythm problem		509	3.81 (3.73–3.90)	0.043	-0.198	0.997	-
4: Compound problems		509	3.77 (3.62–3.92)	0.077	0.872	0.820	-
Medium flexibility							
1: Good sleep		509	3.76 (3.71–3.81)	0.025	-	-	-
2: Sleep debt		509	3.71 (3.65–3.77)	0.031	1.288	0.571	-
3: Sleep-wake rhythm problem		509	3.66 (3.62–3.69)	0.018	3.287	0.006	1 > 3
4: Compound problems		509	3.54 (3.47–3.60)	0.033	5.364	< 0.0001	1 > 4
Mild flexibility							
1: Good sleep		509	3.65 (3.59–3.72)	0.031	-	-	-
2: Sleep debt		509	3.61 (3.54–3.68)	0.036	0.846	0.832	-
3: Sleep-wake rhythm problem		509	3.58 (3.55–3.62)	0.020	1.922	0.220	-
4: Compound problems		509	3.47 (3.40–3.54)	0.036	3.765	0.001	1 > 4
Inflexibility							
1: Good sleep		509	3.42 (3.32–3.53)	0.052	-	-	-
2: Sleep debt		509	3.67 (3.55–3.80)	0.063	-3.033	0.014	1 < 2
3: Sleep-wake rhythm problem		509	3.55 (3.46–3.64)	0.045	-1.867	0.244	-
4: Compound problems		509	3.53 (3.40–3.66)	0.067	-1.251	0.595	-

df; Degrees of Freedom, Emmean; Estimated Marginal Mean, p; p-value, SE; Standard Error, t; t-value, 95%CI; 95% Confidence Interval.

## Discussion

The primary objective of this study was to examine the characteristics of sleep problems (Trisleep) and psychological flexibility (Hexaflex) among young adults. Additionally, it sought to explore the associations of Trisleep and Hexaflex with their physical and mental well-being.

### Classification of Trisleep and Hexaflex clusters

Cluster analysis was conducted to categorize Trisleep and Hexaflex profiles, which revealed four distinct clusters. Trisleep clusters were identified as types of good sleep, sleep debt, sleep–wake rhythm problems, and compound problems; the findings aligned with those of previous studies. Sleep–wake rhythms and sleep debts are commonly observed during university students^39–41^. Insomnia increases with age due to changes in sleep architecture^42,43^. The findings of this study are likely a part of a more compound constellation of sleep problems rather than an isolated issue for university students experiencing insomnia. The current study found sleep–wake rhythm problems to be the most prevalent, aligning with previous findings^41^. In addition, in a cohort study of teenagers, 20% of them were found to be short sleepers (≤ 6 h) at baseline, which increased to 25% after a year^44^. In this study, sleep debt and compound sleep problem types together accounted for 30.7% of the total sleep debt, consistent with previous studies^4,41^. Furthermore, sleep problems in university students have been well documented. A large-scale study of 4,768 undergraduates reported that the eveningness chronotype was associated with poorer subjective sleep quality (OR = 1.671, 95% CI 1.414–1.975), longer sleep latency (OR = 1.436, 95% CI 1.252–1.647), shorter sleep duration (OR = 2.149, 95% CI 1.506–3.067), lower habitual sleep efficiency (OR = 1.702, 95% CI 1.329–2.180), greater daytime dysfunction (OR = 1.602, 95% CI 1.412–1.818), and overall poor sleep quality (OR = 1.866, 95% CI 1.586–2.196), compared with the neutral chronotype^41^ These findings suggest that evening chronotypes may be more likely to experience insomnia-related symptoms.

Contrary to our expectations, no prominent feature was observed between the Hexaflex clusters, aside from variations in the overall size of the pentagonal representation. This study is among the first to explore Hexaflex comprehensively, although the factors influencing these results remain unclear. One possibility is that enhancing a single Hexaflex variable among university students may indirectly improve overall psychological flexibility. This suggests the importance of individualized interventions targeting specific processes within the ACT framework rather than addressing all variables equally. Further research is required to clarify this point.

### Impacts of both sleep problems and psychological flexibility on physical and mental well-being

The influence of Trisleep and Hexaflex on physical well-being, examined in this study, revealed that only Trisleep was significantly associated with physical well-being. Individuals with compound sleep problems are more likely to report severe physical symptoms. Previous studies have shown that insufficient sleep or insomnia exacerbates physical pain, which is consistent with the current findings^3,8,9^. Cognitive behavioral therapy for insomnia (CBT-I) has been shown to effectively reduce pain severity and pain-related disability, and is superior to pain-focused therapies such as CBT for pain in many measurements^45^. Additionally, a secondary analysis of randomized control trials on generalized anxiety disorder (GAD) interventions found that daytime dysfunction improvements were predominantly related to insomnia improvements, but not GAD-related symptoms^46^. These findings underscore the critical role of sleep improvement in alleviating physical symptoms.

In contrast, the influence of Trisleep and Hexaflex clusters on mental well-being revealed that significant interaction effects. Sleep problems and psychological inflexibility were associated with lower mental well-being, aligning with previous research^5–7,19,47^. However, the interaction effects suggested that Hexaflex may play a more critical role in mental well-being than Trisleep, considering its consistent impact. These results imply that, while both psychological (Hexaflex) and biological (Trisleep) factors influence mental well-being, improving mental health may be more important for young adults.

Studies on ACT interventions for insomnia have focused on helping individuals achieve psychological distancing from insomnia symptoms. Both ACT-I and CBT-I have demonstrated efficacy in improving sleep^48,49^. Conversely, CBT-I has not shown great improvements in quality of life^49^. In light of these considerations and the results of this study, targeting only sleep improvement or psychological flexibility may be insufficient to achieve broader functional improvements, including enhancing physical and mental well-being.

Finally, by incorporating sleep–wake rhythms and sleep debt, along with insomnia symptoms, this study provides a comprehensive understanding of sleep problems in young adults. Notably, individuals with insomnia alone were not identified, whereas those with sleep–wake rhythm or sleep debt issues showed poorer mental and physical well-being than those without sleep problems. Although the effects of CBT-I on insomnia symptoms in young adults have been reported^50^, this study highlights the necessity of addressing both biological and psychological factors during young adulthood to achieve optimal outcomes.

### Limitations of the study

Considering that the present study was conducted on healthy university students, the results may likely differ if psychiatric patients or patients with sleep problems were targeted. In addition, it is uncertain whether these results are applicable to other age groups. Furthermore, the study did not include an assessment of social well-being (e.g., absenteeism from school, academic performance, interpersonal relationship etc.) or mental health issues such as depression and anxiety. A comprehensive study including these aspects would perhaps provide more wide-ranging findings.

The present study operationalized sleep quality using the AIS rather than the Pittsburgh Sleep Quality Index (PSQI) because the AIS measures both nocturnal and daytime symptoms and has a relatively small number of items. Although the AIS is strongly correlated with the PSQI (*r* = .81)^27^, our assessment may not fully capture the multidimensional nature of sleep quality. Future research should incorporate additional measures that more comprehensively assess this construct.

Likewise, this study used the APQ, but not the Acceptance and Action Questionnaire-II (AAQ-II), to measure acceptance. Because the APQ was developed specifically for the Japanese context, it has been extensively examined for reliability and validity, and the AAQ-II shows a high correlation with the CFQ in Japan (*r* = .83)^51^, making it difficult to discriminate between the constructs. Whereas the APQ shows a more modest correlation with the CFQ defusion subscale (*r* = .35)^32^, allowing for better construct discrimination.

Moreover, the study did not collect information on participants’ history of sleep-related treatment or engagement in ACT. Without this information, it remains unclear whether prior therapeutic experiences influenced sleep patterns or psychological flexibility. Future studies should assess these factors to better contextualize the observed associations.

Sex distribution in the present sample was imbalanced, with a greater number of female participants. Although supplementary analyses revealed minimal sex differences in the scales, this imbalance may still limit the generalizability of the findings. Future studies should consider employing recruitment strategies such as stratified sampling or matching procedures to obtain a more balanced sex distribution and to further clarify potential sex-related influences on sleep and psychological flexibility.

## Conclusion

The results of this study indicate that both biological and psychological factors play a crucial role in the physical and mental well-being of the young population. Specifically, physical well-being was strongly associated with sleep conditions, suggesting that interventions targeting sleep regulation may be particularly beneficial. In contrast, psychological well-being was associated with both sleep conditions and psychological flexibility, with psychological flexibility showing a stronger association, suggesting that interventions aimed at enhancing psychological flexibility may be especially effective. These findings highlight the importance of assessing potential sleep problems and psychological inflexibility when addressing declines in well-being among young people. Overall, a dual-focus approach targeting both daytime psychological processes (ACT) and sleep quality (CBT-I) could be practically applied in youth mental health programs to promote holistic well-being.

## Supplementary Information

Below is the link to the electronic supplementary material.

Supplementary Material 1

## Data Availability

The data that support the findings of this study are openly available in Zenodo at[https://doi.org/10.5281/zenodo.15043139](https:/doi.org/10.5281/zenodo.15043139).
