# Supplementary material for: Impact of sleep and psychological flexibility on young adults’ physical and mental well-being
Source: Sci Rep. 2026 May 20;16:23053. doi: 10.1038/s41598-026-53737-4 (PMC13392371; doi:10.1038/s41598-026-53737-4)
Supplement: Supplementary file 1 — Supplementary Material 1 [file 41598_2026_53737_MOESM1_ESM.docx]

Supplementary 1. The reliability and validity of each scale

| Scale | Internal consistency | validity |
| --- | --- | --- |
| Somatic Symptom Scale-8 (SSS-8) | Cronbach’s α = .86 | Strong correlations with EQ-5D (ρ = −.54) and POMS subscales (ρ = .51–.61) |
| Subjective Well-Being Scale (SWBS) | Cronbach’s α = .84 | Strong correlations with related constructs: marital satisfaction (men: r = .34, women: r = .46, p < .001), workplace satisfaction (men: r = .56, women: r = .37, p < .001) |
| Athens Insomnia Scale (AIS) | Cronbach’s α = .88 | Strong correlations with PSQI-J (r = 0.81, 95% CI: 0.78–0.84) and ISI-J (r = 0.85, 95% CI: 0.82–0.87). |
| Biological Rhythms Interview of Assessment in Neuropsychiatry (BRIAN) | Cronbach’s α = .84 | Moderate correlations with MEQ: total (r = 0.38), Factor 1 (r = 0.23), Factor 2 (r = 0.45), Factor 3 (r = 0.26) (all p < .05 except Factor 1 p = 0.079) |
| Sleep Debt Index (SDI) | -（no reported） | Using the SDI allows the U-shaped association between sleep duration and depressive symptoms to be linearized. |
| Cognitive Fusion Questionnaire (CFQ) | Cronbach’s α = .68 | CFA: GFI = .91, AGFI = .88, RMSEA = .08 (adequate fit)  Weak but significant positive correlations with activation of BADS (r = .22), observing (r = .23) and describing (r = .20) of FFMQ. Weak negative correlations with PSWQ (r = −.19) and AAQ-II (r = −.19). No correlation with RRQ (r = −.09). |
| Values of Younger Ages Scale (VOYAGE) | McDonald’s ω =.89 | CFA: CFI = .92, SRMR = .04, RMSEA = .08 [95% CI: .07–.08] (adequate fit).  Moderate-strong correlations with AAQ-II (r = .31), EROS (r = .61), SWBS (r = .61) (all p < .01) in university sample. |
| Three Senses of the Selves Questionnaire (TSSQ) | Cronbach's α = .78 and .72, respectively. | Moderate-strong correlations with observing (r = .47) and describing (r = .26) of FFMQ, decentering of J-EQ (r =. 52). (all p < .01) |
| Acceptance Process Questionnaire (APQ) | Cronbach’s α =.86. | CFA: CFI = .96, TLI = .94, RMSEA = .07, SRMR = .05 (adequate fit).  Total score showed weak correlations: AAQ-II (r = −.27), defusion of CFQ (r = .25); activation of BADS (r = .25); nonreactivity of FFMQ (r = .31) (all p < .001). |

Note. AAQ-II; Acceptance and Action Questionnaire–II, AGFI; Adjusted Goodness of Fit Index, BADS; Behavioral Activation for Depression Scale, CFA; Confirmatory Factor Analysis, CFI; Comparative Fit Index, CI; Confidence Interval, EQ-5D; EuroQol 5 Dimensions, EROS; Environmental Reward Observation Scale, FFMQ; Five Facet Mindfulness Questionnaire, GFI; Goodness of Fit Index, ISI-J; Insomnia Severity Index–Japanese version, J-EQ； Japanese Experience Questionnaire, MEQ; Morningness–Eveningness Questionnaire, POMS; Profile of Mood States, PSQI-J; Pittsburgh Sleep Quality Index–Japanese version, PSWQ; Penn State Worry Questionnaire, RMSEA; Root Mean Square Error of Approximation, RRQ; Rumination–Reflection Questionnaire, SRMR； Standardized Root Mean Square Residual, TLI; Tucker–Lewis Index.


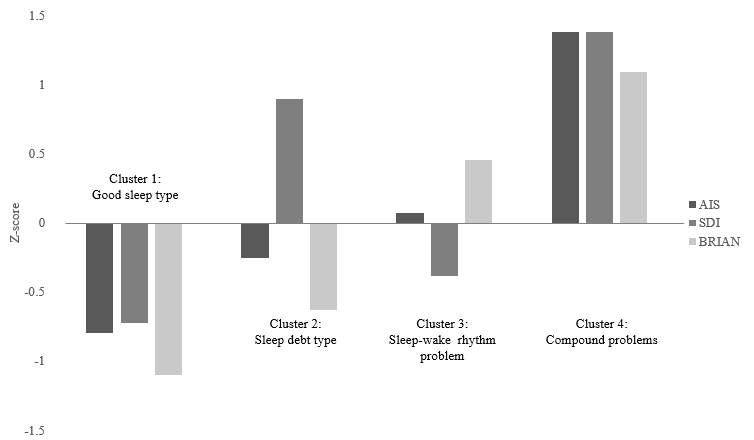


Supplementary 2. Results of Hierarchical Cluster Analysis Based on Trisleep Variables. AIS; Athens Insomnia Scale, BRIAN; biological rhythm was a Biological Rhythms Interview of Assessment in Neuropsychiatry, SDI; Sleep debt index.


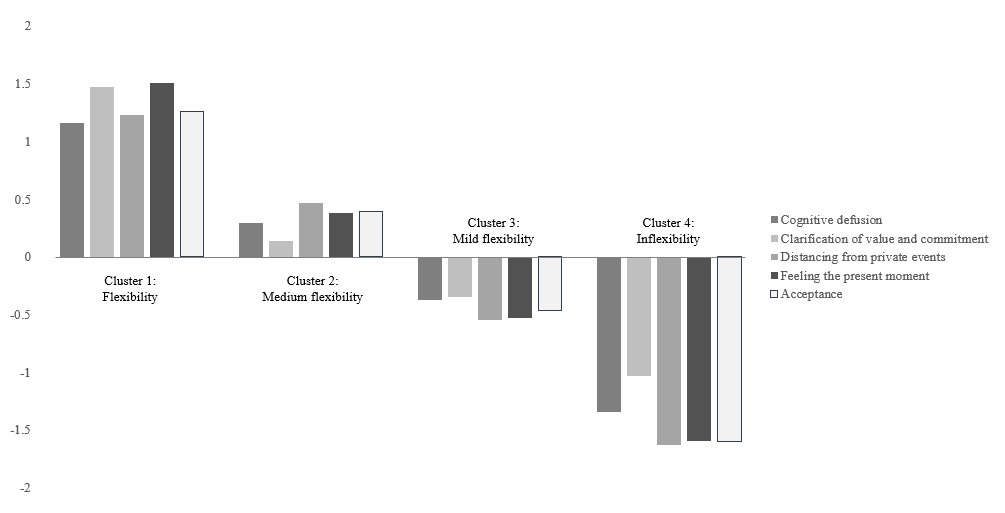


Supplementary 3. Results of Hierarchical Cluster Analysis Based on Hexaflex Variables. The valuables of cognitive defusion was a domain of Cognitive Fusion Questionnaire (CFQ), Clarification of value and commitment was a domain of Values of Younger Ages scale (Voyage), distancing from private events and feeling the present moment were domains of Three Senses of the Selves Questionnaire (TSSQ), and acceptance was total score of Acceptance Process Questionnaire (APQ).
